# Extracts from the Annual Report of the Surgeon-General of the United States Navy

**Published:** 1887-12

**Authors:** 


### EXTRACTS FROM THE ANNUAL REPORT OF THE SURGEON-GENERAL OF THE UNITED STATES NAVY.

1887.

NAVAL HOSPITALS.

The request for increased appropritions for the preservation and repair of the several naval hospitals must be again repeated. These buildings have been in service for many years, and the work of restoring roofs and improving the systems of plumbing and drainage is urgently demanded.

At the naval hospital, Yokohama, Japan, the privilege accorded to the consul-general of sending sick and disabled men from the United States merchant service to be cared for in the hospital has been exercised throughout the year.

NAVAL SANITARIUM, PENOBSCOT BAY.

At the last session of Congress the sum of $50,000 was appropriated "for the construction of a naval hospital and sanatarium and wharf for landing on Widow's island, Penobscot bay, Maine, said sum to be in full for all expenses of erecting and furnishing said sanitarium, including all necessary improvements on the island."

The building will be completed, weather permitting, by December 15, 1887.

MUSEUM OF HYGIENE.

On July 1 the Museum of Hygiene was removed to a large aud commodious building in New York avenue, near the Navy Department, where it is well established, with room for its increasing collection.

Dr. Turner's report is presented herewith.

Tentative experiments have been made at the museum upon the steel used in the new guns for the Navy in order to determine the chemical value of the steel.

These examinations have been limited to the determination of the amount of carbon, silicon, phosphorus, and sulphur existing in the samples presented. The investigation is interesting and important, as the presence of these bodies is known to affect the physical properties of steel.

MEDICAL CORPS OF THE NAVY.

The condition of the Medical Corps of the Navy, which was represented to you last year and commended in your yearly report to the attention of Congress, urgently calls for legislative action. The numbers are still diminishing. There are now twelve vacancies for assistant surgeons. The loss of members in the active list of the corps during the year amounted to nine officers; and only six assistant surgeons were appointed, one of whom has since resigned to enter the Medical Department of the Army.

A bill to improve the condition of this department of the Navy has been prepared to be submitted for your approval on the meeting of Congress.

INSANE OF THE NAVY.

There were sixty-nine patients belonging to the Navy treated in the Government Hospital for the Insane, in the District of Columbia, for the year ending September 30, 1887.

| | | |
|---|---|---|
| Remaining in hospital September 30, 1887 | 60 | |
| Admitted during the year ending September 30, 1887 | 9 | |
| Total under treatment | 69 | |
| Discharged during the year: | | |
| Recovered | 6 | |
| Improved | 1 | |
| Died | 4 | |
| | | —11 |
| Remaining in hospital September 30, 1887: | | |
| Officers | 7 | |
| Enlisted | 51— | |
| | | —58 |

In consequence of the inability, from the want of accommodation, of the Asylum for the Insane at Napa, Cal., to receive any longer the insane patients from the Navy, the Bureau has directed the transfer of all of the insane of the Navy on the Pacific coast to the Government asylum near Washington, D. C.

### REPORT UPON THE MUSEUM OF HYGIENE.

By T. J. Turner, M. D., Director of the Museum.

Herewith is transmitted the second report upon the Museum of Hygiene under its present direction.

The library has not been increased to any great extent, and the want of space for the exhibition of sanitary appliances has retarded inventors from forwarding such new inventions as present the more recent scientific results in the mechanics of sanitation. The greater number of such mechanical devices now on exhibit relate to house sanitation.

The clerical duty involved in the daily routine of the museum has not permitted personal work upon many questions in hygiene demanding experimental investigation. The index catalogue of the library has been attended to so far as possible. The services of a clerk to perform the duty of a librarian has become a necessity. The increase of the chemical work demands an increase in the pay of the skilled assistant now employed in the laboratory.

The removal of the museum in its entirety to the present location necessarily involved the arrest of all work then on hand, and all projected work is still in abeyance, until the new arrangement of the establishment shall have been perfected to such a degree that the usefulness of the museum may not be impaired. The number of visitors to the museum has steadily increased, and there is much of interest displayed by plumbers, sanitary engineers, and architects in the collection and the library.

### AIR.

During the past year there has been but little advance made in the chemical examination of the air for sanitary purposes. The determination of the amount of $CO_2$ still remains as the most convenient factor upon which organic impurity is assumed. Modifications of the apparatus for the determination of $CO_2$ by Pettenkofer's method frequently appear in the illustrated sanitary journals. It is a known fact that the amount of $CO_2$ in the air, usually stated as 4 volumes per 10,000, is slightly in excess of the real amount as determined by more recent research. This excess, however, is not so great as to impair the value of the $CO_2$ estimation as the measure of respiratory impurity. No new investigations as to the organic impurities in the air have come under observation.

### WATER.

The well-decided facts of the water carriage of the poison of cholera, and that in all the ports visited by our naval vessels the water supply to vessels is more or less to be suspected, and that following the use of distilled water on shipboard there has been a notable decrease of abdominal fluxes, even in some instances to total immunity from such disease action, it seems therefore advisable that some general order regarding the use of distilled water on shipboard should be issued. The constant iteration of this may at last awaken some naval authority to its importance. It is well known that the tests required from the medical officer, hurriedly made when "the water boat is alongside," are of no decided value in determining the safety-mark of potability.

There has been added to the exhibit a Baird's distiller and aërator. This apparatus is, so far as can be ascertained, the most useful of its kind for use at sea, and it is equally adapted for such purpose on shore. It can readily be attached to an ordinary kitchen range, and would afford an ample supply for all household purposes.

### FOOD.

The following table of the composition of foods relates to the ration, and is but complemental to the note on foods in the first report:

*Composition of foods.*

| Articles. | Water. | Albuminoids. | Fats. | Carbohydrates. | Salts. | Other constituents. |
|---|---|---|---|---|---|---|
| Bread | 8. | 15.6 | 1.3 | 73.4 | 1.7 | |
| Biscuit | 40. | 8. | 1.5 | 49.2 | 1.3 | |
| Soft | 15. | 11. | 2. | 70.3 | 1.7 | |
| Corn-meal | 14. | 11.1 | 8.1 | 65.1 | 1.7 | |
| Corn-hominy | 13.5 | 9.9 | 6.7 | 64.5 | 1.4 | Cellulose, 4. 0. |
| Oat-meal | 15. | 12.6 | 5.6 | 63. | 3. | |
| Rye | 15. | 8. | 2. | 69.5 | 1.8 | Sugar, 3. 7. |
| Wheat | 13.56 | 12.42 | 1.7 | 70.55 | 1.79 | |
| Pork (salt) | 44.1 | 26.1 | 7. | -------- | 22.8 | |
| Beef (salt) | 49.1 | 29.6 | .2 | -------- | 21.1 | |
| Roast beef | 54. | 27.6 | 15.45 | -------- | 2.95 | |
| Canned mutton | (*) | (*) | -------- | -------- | -------- | |
| Chicago corned beef | 40. | 40. | 15. | -------- | 5. | |
| Brawn | (†) | (†) | -------- | -------- | -------- | |
| Ham | 27.8 | 24. | 36.5 | -------- | 10.1 | |
| Bacon | 15. | 8.8 | 73.3 | -------- | 2.9 | |
| Sausage | (*) | (*) | -------- | -------- | -------- | |
| Fish (fresh): From | 75. | 10. | 1.1 | -------- | 1. | |
| Fish (fresh): To | 85. | 19. | 13.8 | -------- | 1.8 | |
| Fish, dried (cod) | 78. | 18.1 | 2.9 | -------- | 1. | |
| Beans | 14.76 | 24.27 | 1.61 | 56.10 | 3.26 | |
| Pease | 15. | 22. | 2. | 53. | 2.4 | |
| Rice | 10. | 5. | .8 | 83.2 | .5 | |
| Apples, fresh | 86.28 | .08 | -------- | 6.45 | .3 | Malic acid, .08; woody fiber, 3.8. |
| Peaches, fresh | 85. | .46 | .61 | 1.6 | .5 | Cane sugar, 7.6—to be added to carbo-hydrates. |
| Raisins | -------- | 4.1 | -------- | 68. | 2.3 | |
| Fresh currants: | | | | | | |
| Red | 85.27 | .68 | -------- | 6.44 | .57 | |
| White | 84.8 | .30 | -------- | 7.69 | .56 | |
| Figs | 24.94 | 13.3 | -------- | 48.9 | 1.77 | |
| Dates | 21.6 | 10.93 | -------- | 56.41 | 1.5 | Cane sugar, 56.41; oil, .19; pectin, 6.99; woody fiber, 2.38. |
| Butter | 6. | .3 | 91. | -------- | 2.7 | |
| Tomatoes | 89.8 | 1.4 | -------- | 6.7 | .8 | Cellulose, pectose, 1.3. |
| Coffee | .36 | 12.03 | 8.3 | 1.84 | 5.17 | Cane sugar, 1.84; caffeine, 1.06; gum, tannin, 26.28; cellulose, 44.96. |
| Tea (black) | 11.56 | 15.55 | -------- | -------- | 5.82 | Tannin, 15.24; theine, 2.53. |
| Tea (green) | 9.37 | 24.39 | -------- | -------- | 5.38 | Tannin, 18.69; theine, 2.79. |
| Cocoa | 6.12 | 18. | -------- | 13. | 3.6 | Cocoa butter, 50. |
| Sugar | 3. | -------- | -------- | 96.5 | 3.6 | Cane sugar, 96.5. |
| Pickles | (*) | (*) | -------- | -------- | -------- | |
| Molasses | 27.3 | -------- | -------- | 67.4 | 2.6 | Cane sugar, 47; extractives, 27; glucose, 29.4. |
| Vinegar | 87.6-96.3 | -------- | -------- | -------- | -------- | Acetic acid, 3 to 10; extractive, 1.7 to 2.4. |
| Beef (fat) | 45.6 | 15. | 84.8 | -------- | 4.6 | |
| Mutton (fat) | 39.7 | 11.5 | 45.5 | -------- | 3.5 | |
| Veal (fat) | 62.3 | 16.6 | 16.6 | -------- | 4.5 | |
| Pork (fat) | 38.6 | 10.5 | 49.5 | -------- | 1.4 | |
| Poultry | 74. | 21. | 3.8 | -------- | 1.2 | |
| Vegetables | (*) | (*) | -------- | -------- | -------- | |

* Composition variable. † Same as bacon.

Some discussion upon the bulk of the ration has been had of late, and the tendency is toward decreasing the bulk, as compared with weight, as a saving in the means of transportation.

Elsewhere the writer, in commenting on the naval ration, has stated that the ration in port represented 32.49 ounces average, and at sea 24.75 ounces average of water-free food. It is to be stated that this result is calculated from the chemical composition of the food, and that long before this weight could be obtained by any process of dessication physical changes occur, rendering the food unfit for nutritive purposes. It is a matter of every-day observation on shipboard that dried vegetables especially do not regain their original sapidity when treated with fresh water, added for the purpose of restoring them to their normal condition.

The fashion for the day in the matter of foods is in the use of the so-called peptonized, malted, soluble, etc., foods. Their use belongs, in a great degree, to the therapeutics. Some experiments have been made in the museum to determine from a physiological basis the nutritive values of foods. As the investigation progressed much was learned of the difficult nature of the examination, wherein the value of each condition had to be determined as influencing any series of observations.

Having in other papers upon matters connected with naval hygiene considered the ration, it is proposed to offer some suggestions concerning the distribution of the food as well as the rearrangement of the meal hours more consonant with the physiology of the digestive act; and, therefore, more conducive to the health of the crew. At present the meal hours are as follows:

Breakfast at 8 A. M.; dinner at 12 noon; supper at 5 P. M., and upon "turning out an additional ration of tea or coffee and sugar." It is evident that the men are from 5 P. M. until 8 A. M., 15 hours, without food, and that the bulk of the food is ingested in the remaining 9 hours of the day. Now, the digestive act is completed, in so far as the finding of the remains of the food in the stomach is concerned in from 2 to 3 hours (Beaumont on Digestion), so that the men in the mid and morning watches, often accused of "loafing" and a want of activity, affected with lassitude and a restless wakefulness, akin to morbid vigilance, are in this condition but giving physiological expression to the wants of the economy requiring food stimulation. This condition is a powerful factor in predisposing to disease, and the cumulative effects are seen in the depressed vitality of the sailor later on in his life.

Considering the variety as well as the bulk of the ration, a proper distribution, from careful study, would be as follows: Dividing the whole ration into 9 parts, assign 3 parts for breakfast, 4 parts for dinner and 2 parts for supper—"that is to say, breakfast to restore the waste, dinner to support during the hours of active toil, and supper to carry on the functions of repair and secretion." (Letheby.) This division, however, does not consider the fact of the loss of sleep in the night watches at sea; but as there is the turning out meal, and a suggestion as to the mid-watch, it is believed to be correct both from a chemical and physical view.

Again, it is known (Smith, "Cyclical Changes in Health and Disease"), that after the indigestion of food, pulsation, respiration and temperature rise, indicating more active functional work in the digestive act, and that this rise occurs within from two to three hours after a meal. It has also been observed that pulsation, respiration and temperature are lowest from 3 to 6 in the morning. It would appear, therefore, that the ration on turning out has a physiological foundation.

Reasoning from these facts, together with the known physical relations of food to work, it is suggested that by shifting the dog watches from 12 to 2, and from 2 to 4, the following arrangement of the meal hours could be made which does not interfere with the routine work of the ship: Continuing the ration as directed by law upon turning out, to have breakfast at 8 A. M., dinner at 2 P. M., and supper at 8 P. M., reserving for the mid-watch, as they go on duty, a portion of the ration of tea or coffee and a biscuit.

Thus the food of the sailor is served out more in accord with physiological requirement, the health and energy of the men are conserved and correlated, there is more immunity from disease-influence, and so their efficiency for work is kept at its best standard.

As not foreign to this subject, the experience of a British officer at Gibraltar should be mentioned. Giving his troops a supper at 8 P. M., it was observed that a manifest decrease in the number of cases of drunkenness followed this salutary measure. The stimulus of food had supplied the desire for rum.

There are but few instances of any variation from the ancient methods of cooking meals on shipboard. The "coppers" still retain the name, although that metal has long been disused in the construction of the galley. The preference of the writer has already been given to the "improved galley" for use in ships of war, but this short sketch would be incomplete without some hints as to the personnel of the cooking force.

To secure economy, cleanliness, comfort, and so aid in the maintenance of the health of the crew, the following suggestions are offered:

As one ration in four may be commuted, and as the money value thereof is expressly directed to be expended "for the sole purpose of affording a means of adding to and increasing the variety of food provided by the Government"—establish the rating of ship's steward—who should be directed to make purchases, when in port, of such articles as the messes may require; to post, weekly, for the information of the crew, the price current of the articles purchased, and be held responsible for the moneys intrusted to his charge for such purposes. Retain the present billet of ship's cook, abolish the present system of mess cooks, and make a rating of assistant cooks, in the proportion of one to every thirty men of the ship's complement. Build a pantry, doing away with the cumbersome mess chests that now lumber up the decks, stow in the pantry all mess gear—"pots, kettles and pans," when not in use. Let the assistant cooks keep the pantry clean, spread the mess gear for meals, gather up the same after meals, cleanse and re-stow them in the pantry, attend to the serving out of the ration, the preparation of the food, as well as its distribution at the appointed meal hours, etc.

These suggestions are departures from old customs, but are warranted by the advancing study of naval hygiene.

In the matter of poisoning from canned food, the following may prove instructive:

*The results of the micro-chemical observations made at the museum upon the following articles received from the hands of Passed Assistant Surgeon C. W. Deane, U S. Navy.*

Two wide-mouthed glass bottles—one stated to contain "canned ham," suspected to have caused symptoms of poisoning, and one stated to contain a sample, unused, from the same invoice as the suspected ham.

It was also stated that the symptoms of poisoning occurred about two and a half hours after the ingestion of the suspected ham.

A portion of this meat, after twenty-four hours' immersion in alcohol sp. gr. .820, was treated with HCl (one part to ten parts of water), in a porcelain capsule, over a water-bath, for two hours, at a temperature of 37.5°C., and then filtered; a portion of the filtrate was examined by the usual tests, and gave no reaction for any metallic substance.

From the odor there was no evidence of any putrefactive change. The fat and muscular tissue, however, were readily separable, the connective tissue easily breaking down under slight pressure of a glass rod. The muscular fiber also readily separated into small bundles, recalling the "boiled rope yarns" of canned meats, so well known to messes on shipboard. Morache ("Traité d'Hygiène Militaire," 1886) states that the frequent accidents which so often follow the ingestion of salted meats badly conserved have no other origin than from the formation of septic alkaloids from the albuminoid principles of the meat. Accepting these appearances as those of a retrogressive change and as evidences of the want of the preservation of the food by the "canning method," the following experiments were instituted for the isolation and detection of such bodies:

A portion of the acid solution was treated with a solution of *sodic hydrate* until slightly alkaline. A slight precipitate appeared, which, under the microscope, was found to be composed of minute crystals of sodium chloride.

Another portion of the acid solution was treated by ammonia water and *ether;* the ethereal solution was separated and allowed to evaporate spontaneously, then treated with a solution of osmic acid, and examined under the microscope; fat globules only were apparent.

Another portion of the acid solution was treated by ammonia water and *chloroform;* the chloroform solution separated and allowed to evaporate spontaneously, and the residue examined under the microscope: crystals of ammonium-chloride only were found.

Another portion of the acid solution was treated by ammonia water and *amyl-alcohol;* the amyl-alcohol solution was separated and evaporated over a water-bath, the residue examined under the microscope: crystals of sodium-chloride only were found.

Another portion of the acid solution was treated by ammonia water and *dilute alcohol;* the dilute alcoholic solution obtained was evaporated over a water-bath and the residue examined under the microscope: crystals of ammonia-chloride only were found.

A watery solution, obtained by digesting a part of the suspected matter in distilled water over a water-bath for two hours, treated with ether to remove fatty matters, and the separated *aqueous solution* examined, after evaporation by a gentle heat, under the microscope, presented crystals of sodium-chloride, and no trace of any other matter.

Upon testing a portion of this aqueous solution by potassic-mercuric iodide for organic matter, there was no reaction.

A portion of the aqueous solution was rendered acid by dilute sulphuric acid; to a part of this solution was then added a few drops of a freshly prepared solution of potassic-ferridcyanide, and then tested by a dilute solution of ferric-chloride—no reaction showing that any reduction had taken place from the presence of organic matters, especially the ptomaines, was evident.

No evidence of trichina were found. The microscopical examination of sections of the suspected and unsuspected meat was made by Surgeon C. H. White, U. S. Navy, and no departure from the normal histological appearance in meat was observed.

In so far as the culture-experiments upon these substances have progressed under the management of Passed Assistant Surgeon G. Arthur, U. S. Navy, no generic forms of zymogenic bacteria have been found, after "cultivation" at a constant temperature of 35°C. for five days in sterilized test-tubes.

From the facts obtained by the chemical examination it is concluded—

1st. That the phenomena presented in these cases of poisoning can not be ascribed to any *inorganic poison*.

2d. That the examination for the alkaloidal bodies, resulting from slow chemical changes in the suspected meat, does not reveal their existence by methods used for their isolation. In other words, no *organic poison* was detected by the methods described.

These processes were arranged to capture, so to speak, any of these bodies by their solubilities or insolubilities in the different menstrua used, and the processes are alike applicable to the use of other acids, organic or inorganic, and to other menstrua, such as benzole, petroleum-ether, etc., in like examinations.

Although the entire examination has resulted as stated, it is deemed proper to present all the processes used from which the conclusions have been obtained.

CLOTHING.

An extended examination upon the clothing furnished the crews of naval vessels as to its hygienic value was inaugurated and placed under the charge of Passed Assistant Surgeon G. Arthur, U. S. Navy. It has not been completed to the extent at first lined out, but enough has been determined to make questionable some of the published results of the investigations, heretofore accepted as positive, of Coulier and Hammond.

LIGHTING.

Of the hygienic value of the electric light on shipboard and its manifest advantages no reports have been received at the museum.

DISINFECTANTS.

Examination of various proprietary articles of this character reveals the fact that they are mixtures of well-known disinfectants.

An investigation as to the value of iodoform as an antiseptic was commenced and abandoned for want of the necessary time needed for such investigation.

The usual chemical, microscopic and physical examinations have been carried on as required, and this report would be incomplete if there was failure to acknowledge the able aid rendered the Director by Surgeon C. H. White and Passed Assistant Surgeon G. Arthur, U. S. Navy, on duty at the museum.

GENERAL CONSIDERATIONS.

Upon the tag marking the price of peace, in our modern civilization, is emblazoned the trade-mark of war — a gun; and whilst attention has been directed more toward the management of masses of men, observant medical men have quietly been considering the results of the first contact of bodies armed with modern instruments of warfare. Granting two opposing bodies — armed with the long-ranged rifled gun, the rapid firing machine gun and the magazine rifle in the hands of the trained soldier — the military medical officer is, of necessity, brought to the front to render the first aid to the wounded. And here the ambulance system should be considered in all its relations. It is not proposed to enter upon a study of the methods suggested for the care of the health and comfort of the wounded, but some remarks as to the status of the medical military officer arise in this connection, and are pertinent to this report. It is fair to assume that the number of wounded requiring aid will be vastly increased over the number of wounded in previous conflicts, and no ambulance apparatus for rendering such aid, be it ever so simple, can be carried in such number as to hamper in the least degree the movements of the opposing forces. Even now the propriety of incumbering the soldier with a few bandages is a subject of discussion, for the drift in all these matters is to reduce to the minimum all the incumbrances to rapid movements. All this applies to movements on land, yet the difficulties are multiplied for the naval medical officer. It needs no stretch of the imagination to depict a landing party, covered by the guns of a vessel, handled by trained men, exposed to a fire from the shore from equally trained gunners. To such a fire the medical officer is as much exposed as his leader. Nor is this all; in times of peace he has to be prepared for war — an incident in the naval life. The steady additions to the etiology of disease requires him to be always on the alert, to practically apply his knowledge, under the varying conditions of climate and shipboard life, to the prevention and arrest of disease, by attacking its causation.

